# The moderating role of caring for grandchildren in the relationship between functional abilities and depression of aged parents in rural China

**DOI:** 10.3389/fpubh.2025.1621954

**Published:** 2025-07-29

**Authors:** Haijun Hao, Ruonan Li

**Affiliations:** ^1^School of Public Administration (School of Philanthropy), Shandong Technology and Business University, Yantai, China; ^2^Department of Social Welfare, Jeonbuk National University, Jeonju, Republic of Korea

**Keywords:** functional abilities, depression, aged parents, caring for grandchildren, rural China

## Abstract

**Introduction:**

This study investigates the impact of functional abilities on depression among rural older adults in China, with a focus on the moderating role of caregiving for grandchildren.

**Methods:**

Using data from the 2018 China Health and Retirement Longitudinal Study (CHARLS), 4,366 valid responses were analyzed. Hierarchical regression was employed to examine the relationships between IADL, caregiving, and depression.

**Results:**

The findings indicate that higher functional ability is associated with lower depression levels (*β* = −0.402, *p* < 0.001), and caregiving for grandchildren significantly moderates this relationship (*β* = −0.203, *p* < 0.001). Specifically, older adults with higher functional ability who care for grandchildren experience greater reductions in depression.

**Discussion:**

The study concludes that caregiving for grandchildren buffers the negative effects of functional abilities decline on depression, highlighting the mental health benefits of intergenerational caregiving in rural China.

## 1 Introduction

Globally, populations are aging rapidly, driven by rising life expectancy and falling fertility rates (1). This demographic trend is particularly evident in China, where population aging began in the mid-20th century. According to the seventh national census, 264 million people, or 18.7% of China’s total population, are aged 60 and above, and 190 million, or 13.5%, are aged 65 or older (2). The effects of urbanization, particularly the migration of younger individuals to cities, have intensified aging in rural areas. As reported by the Chinese Academy of Social Sciences, the rural population aged 60 and older now comprises 20.04% of the total rural population, while those aged 65 and above account for 13.82%, officially marking China as an “aging society” (3).

As the country with the world’s largest older adult population, China faces significant challenges in addressing the decline in daily living abilities among its older adults. Aging often leads to a reduction in physical and cognitive capacities, particularly affecting aged parents’ functional ability to carry out activities of daily living (ADLs) and instrumental activities of daily living (IADLs) (4). Notably, among individuals aged 65 and older, 35% suffer from disabilities that impair ADLs and IADLs, making independent living difficult (5). ADLs encompass essential self-care tasks such as dressing, bathing/showering, eating, getting into or out of bed, using the toilet, and controlling urination and defecation, while IADLs include more complex activities like preparing hot meals, shopping for groceries, making phone calls, taking medication, and managing money (6, 7). Maintaining IADLs capabilities is vital for older adults’ independence and overall well-being, allowing them to remain socially engaged and lead healthier lives. Furthermore, functional decline in older adults has been linked to the onset of psychiatric conditions, particularly depression, which exacerbates the challenges faced by the older adult population (8, 9). Addressing both the physical and mental health needs of China’s aging population is thus crucial for fostering a sustainable and supportive care environment.

In rural China, the practice of caring for grandchildren is both widespread and culturally significant (10, 11). According to data from the 2014 China Longitudinal Aging Social Survey (CLASS), 73.29% of older adult parents in rural areas are actively involved in the care of their grandchildren, with this figure continuing to rise (12). This role is a vital aspect of daily life for older adults in rural areas, offering a sense of purpose and social engagement (13). However, the physical and mental health of these aged caregivers is increasingly at risk due to the natural decline in their functional abilities over time. Functional decline, in ADLs and IADLs, which includes tasks such as dressing, bathing, preparing hot meals, shopping for groceries, making phone calls, may hinder their ability to care for their grandchildren effectively. This decline not only affects their caregiving responsibilities but can also lead to increased levels of depression (14). Despite the prevalence of this caregiving role, few studies have thoroughly investigated its impact on the physical and mental well-being of aged caregivers in rural areas. Existing literature has largely overlooked the potential moderating effect of grandchild care on the relationship between functional decline and health outcomes. Understanding whether caring for grandchildren can buffer or exacerbate the effects of functional limitations on depression is crucial for developing comprehensive care strategies (15).

While the relationship between functional abilities and depression among aged parents has been extensively studied (4), no research has yet explored the moderating role of caregiving for grandchildren. This article aims to fill this research gap by examining how functional decline, as measured by the ADLs and IADLs scale, correlates with depression in aged rural caregivers. Furthermore, it explores whether caregiving moderates this relationship, potentially either alleviating or worsening depressive symptoms. Depression levels are assessed using the Center for Epidemiologic Studies Depression (CES-D) scale, which provides insight into the psychological well-being of these aged individuals. Through this analysis, the study contributes to a deeper understanding of the complex interplay between caregiving roles, functional health, and mental health in rural China.

## 2 Literature review

### 2.1 Functional abilities and depression

Older adults, as a distinct demographic, are particularly susceptible to psychological issues due to changes in their physical health, living conditions, and social roles. In recent years, the aging population and the rise of empty nesters have contributed to a notable increase in the incidence of geriatric depression. A meta-analysis revealed that the prevalence of depressive symptoms among older adults in China was approximately 22.6% between 2000 and 2009, rising to 25.5% between 2010 and 2019 among the general older population (16). The ability to perform activities of daily living, a critical indicator of physical health, is strongly associated with the onset of depression in older adults (17). Functional abilities are commonly assessed using the ADLs and IADLs scales. IADLs assess more complex tasks required for independent living, such as shopping, using the telephone, preparing meals, housekeeping, managing medications, traveling independently, and handling finances (18). In some cases, IADLs also include walking long distances, caring for children, navigating the neighborhood, and climbing stairs (19). A decline in functional capacity may lead to feelings of uncertainty and hopelessness about maintaining independence (20).

Extensive research has established a strong relationship between functional abilities and depression (8, 20, 21). In particular, functional impairments are associated with a higher risk of depression in older men compared to women (4, 9). Kiyoshige et al. (22) found that the impact of IADL decline on depressive symptoms varies significantly across age groups, with the strongest effects observed among individuals in their 70s. Furthermore, Zhao et al. (23) demonstrated in a comparative study that rural older adult parents experience functional impairments at an earlier age than their urban counterparts. Numerous studies reinforce the connection between declining functional abilities and heightened levels of depression in older adults (23, 24).

### 2.2 Caring for grandchildren, functional abilities, and depression

Caring for grandchildren can significantly influence the health of older adults (15). However, research findings on the relationship between caregiving and the functional health of aged caregivers vary. Studies on this subject can be categorized into three main perspectives. The first is the “positive effect” perspective, which posits that caregiving has a beneficial impact on older adults’ functional health (25). For instance, Xu (11), using 2011–2013 CHARLS data, found that caregiving reduced hypertension among grandparents, with the health benefits being more pronounced for urban grandparents compared to rural ones. The second category, “negative effects,” argues that caregiving worsens the physical health of aged caregivers and diminishes their functional ability to perform daily tasks (26). Several studies have reported that caregiving negatively affects older adults’ physical health (27, 28). Lastly, the “no effect” perspective asserts that caregiving has no significant impact on the functional health of aged caregivers. For example, Oshio (29), using data from the 14th National Survey of Japan, concluded that caregiving neither benefits nor harms grandparents’ health.

There is no academic consensus on the relationship between caregiving for grandchildren and the mental health of older adults. The first perspective, “positive effects,” suggests that caregiving benefits the mental health of older adults (30, 31). Studies have shown that grandchildren can provide emotional support, reducing loneliness and enhancing mental well-being (25, 32). Improved mental health may also contribute to better physical health outcomes for older adults (33, 34). Burn and Szoeke (35) further argue that caregiving can physically engage older adults, slowing cognitive decline. Additionally, caregiving offers opportunities for older adults to receive financial and emotional support from their children, thereby increasing life satisfaction. The second perspective, “negative effects,” asserts that caregiving can adversely affect the mental health of older adults (28, 36). For instance, Komonpaisarn and Loichinger (37), using survey data from Thai older adults, found that caregiving negatively impacts psychological well-being. Finally, the “no effect” perspective suggests that caregiving has no significant influence on the mental health of older adults (14). Some studies even report that caregiving behaviors have no effect on the overall health or life satisfaction of aged caregivers (29, 38). While limited theoretical models have conceptualized caregiving as a moderator, empirical studies have suggested that the context of caregiving can shape health outcomes (13, 39), justifying further exploration.

### 2.3 Conceptual framework and hypotheses

**Figure 1:**
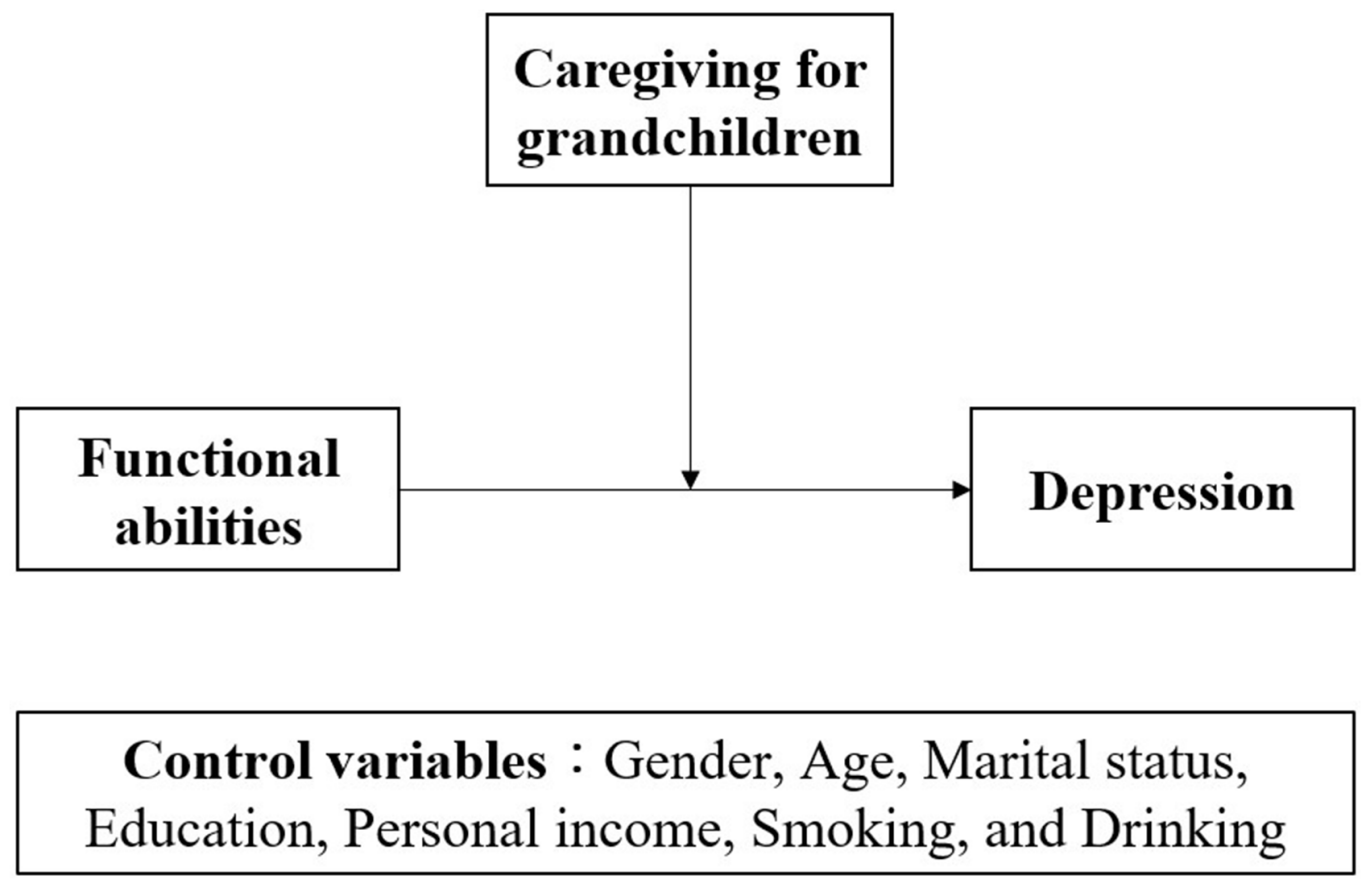
Conceptual framework.

This study proposes a conceptual framework (see Figure 1) where caregiving moderates the relationship between functional abilities and depression. The model is grounded in empirical research but also responds to the current lack of theoretical elaboration on this interaction in Chinese rural contexts.

Based on this framework, the following hypotheses are proposed:

Hypothesis 1 (*H1*): Functional abilities have a positive impact on the depression of rural older adults.

Hypothesis 2 (*H2*): Caring for grandchildren positively moderates the effect of functional abilities on depression in rural older adults.

## 3 Method

### 3.1 Participants and sampling

This study utilizes data from the 2018 China Health and Retirement Longitudinal Study (CHARLS), a nationally representative longitudinal survey designed to reflect the conditions of residents in mainland China aged 45 and older, without an upper age limit. CHARLS aims to provide comprehensive insights into the health, economic, and social conditions of China’s aging population. To ensure representative sampling, the baseline survey covered 150 counties/districts and 450 villages/urban communities across the country, ultimately involving 17,708 individuals from 10,257 households (40). The focus of this study is on rural older adults aged 65 and above who participate in grandchild caregiving. After filtering for invalid responses, a total of 4,366 valid samples were obtained for analysis, ensuring a robust dataset for examining the interplay between caregiving, functional ability, and mental health outcomes among this population. All survey instruments were administered in Mandarin Chinese. The CES-D-10 and IADL/ADL items used in CHARLS have been validated in Chinese populations and demonstrated acceptable reliability (Cronbach’s alpha > 0.75) in previous studies (4, 41).

### 3.2 Ethical considerations

Ethical approval for all the CHARLS waves was granted from the Institutional Review Board at Peking University. The IRB approval number for the main household survey, including anthropometrics, is IRB00001052-11015; the IRB approval number for biomarker collection, was IRB00001052-11014. During the fieldwork, each respondent who agreed to participate in the survey was asked to sign two copies of the informed consent, and one copy was kept in the CHARLS office, which was also scanned and saved in PDF format. Four separate consents were obtained: one for the main fieldwork, one for the non-blood biomarkers and one for the taking of the blood samples, and another for storage of blood for future analyses.

### 3.3 Variables measurement

#### 3.3.1 Dependent variable

Depression was evaluated by the Center for Epidemiologic Studies Depression Scale (CES-D-10) in this study. CES-D-10 using a series of 10 questions designed to measure both negative affect (e.g., feeling useless) and positive affect (e.g., feeling satisfied with life). Respondents were asked to rate how frequently they experienced each emotion over the past year on a four-point scale (1 = Rarely or none of the time; 2 = Some or a little of the time; 3 = Occasionally or a moderate amount of the time; 4 = Most or all of the time). For the purpose of this study, responses were recoded as follows: 0 = “rarely or none,” 1 = “unusual,” 2 = “sometimes or half of the time,” and 3 = “most of the time.” Both negatively worded and positively worded items were combined to create a unified scale, with higher scores indicating more severe depressive symptoms. The total possible score ranged from 0 to 30, with higher values reflecting greater levels of depression (41).

#### 3.3.2 Independent variable

In this study, the independent variable is functional abilities, which refer to reductions in the capacity to engage in ADLs and IADLs due to physical, cognitive, or emotional challenges. The CHARLS questionnaire assessed participants’ functional abilities by examining their self-reported difficulty in performing ADLs and IADLs. ADLs included tasks such as dressing, bathing or showering, eating, getting in and out of bed, using the toilet, and managing bladder and bowel control. IADLs involved more complex tasks necessary for independent living, such as preparing hot meals, shopping for groceries, making phone calls, taking medication, and managing finances (42). Respondents were asked, “Do you have any difficulty with Instrumental Activities of Daily Living?” The response options were as follows: 0 = “I cannot do it,” 1 = “Yes, I have difficulty and need help,” 2 = “I have difficulty but can still do it,” and 3 = “No, I do not have any difficulty.” The responses to these 11 items were summed to create an overall ADLs and IADLs score for each respondent, with a possible range from 0 to 33, with higher scores indicates greater functional abilities for performing daily living activities.

#### 3.3.3 Moderating variable

The moderating variable in this study was caregiving for grandchildren. In the 2018 CHARLS questionnaire, caregiving was measured by asking respondents: “During the last year, did you or your spouse spend time taking care of your grandchildren?” Respondents had three answer options: (1) Yes, (2) No, and (3) I have no grandchild. Participants who answered “yes” were coded as “1 = Yes,” and those who answered “no” as “0 = No” (43). Respondents who indicated they had no grandchildren were excluded from the analysis.

#### 3.3.4 Control variables

We selected control variables based on previous studies examining factors that influence the health status of rural older adults. The control variables included gender, age, marital status, education, personal income, smoking, and drinking (31, 43, 44). Table 1 provides an overview of these control variables, along with the corresponding questions from the 2018 CHARLS questionnaire and their coding for this study.

**Table 1:** Control variables.

Variables	Original questions	Codes
Gender	What is your gender?	0 = Male; 1 = Female.
Age	What is your date of birth on your ID card or household register?	65–85 years old.
Marital status	What is your marital status?	1 = Married; 2 = Divorced\widowed\ never married.
Education	What is the highest level of education you have now (not including adult education)?	0 = Did not go to school; 1 = Elementary school; 2 = Middle school; 3 = High school or above.
Personal income	Did you receive any wage or bonus income in the past year?	Personal income.
Drinking	Ever drinks any alcohol before?	0 = No; 1 = Yes.
Smoking	Have you ever smoked before?	0 = No; 1 = Yes.

### 3.4 Statistical analyses

**Figure 2:**
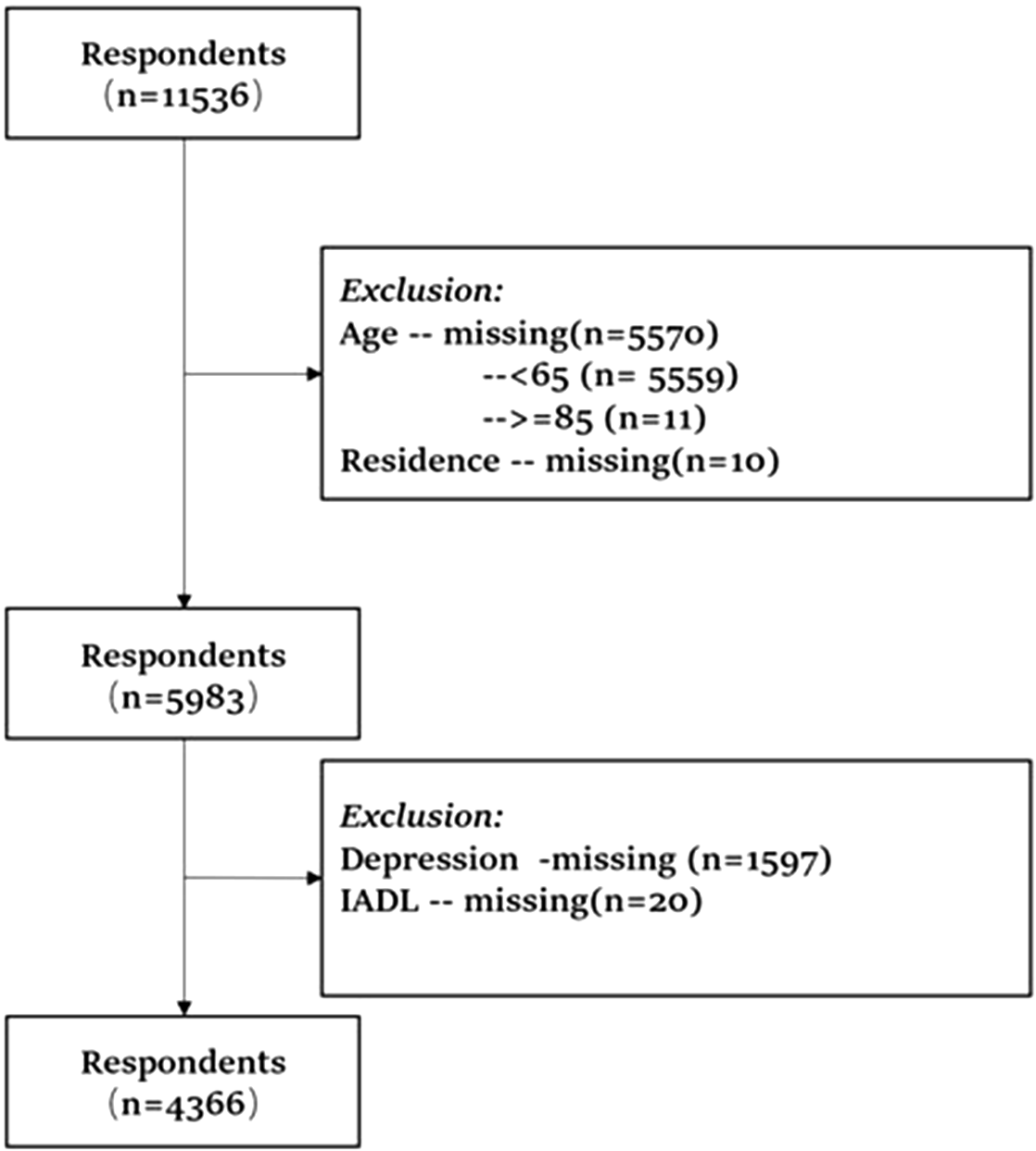
Flow diagram of samples.

In this study, respondents with missing data on age, residence, depression, or IADLs were excluded from the analyses to ensure data integrity and representativeness. The primary analysis focuses on the relationship between functional abilities, caregiving for grandchildren, and depression among older adults aged 65 and above in rural China. Participants without grandchildren or with missing depression data were excluded from the final analysis. This resulted in a total of 4,366 valid responses, as illustrated in Figure 2.

To test the study’s hypotheses, hierarchical regression analysis was employed. In the first step, control variables—including gender, age, marital status, education, personal income, smoking, and drinking—were entered into the model to account for their influence on depression. In the second step, the functional abilities variable was added to examine its direct impact on depression. Finally, in the third step, an interaction term between functional abilities and caregiving for grandchildren was included to assess the moderating effect of caregiving on the functional abilities-depression relationship. Specifically, the interaction term between functional abilities and caregiving for grandchildren was created by centering both variables and multiplying them to reduce multicollinearity, then entered in the final step of the hierarchical regression model. The significance of these interactions was evaluated using the coefficient and *p*-value of the interaction term. Specifically, all analyses were conducted using STATA 17.0.

## 4 Results

### 4.1 Characteristics of the study population

The descriptive statistics for all participants are presented in Table 2. Notably, 67.8% of rural older adults reported providing care for their grandchildren, a percentage significantly higher than those who did not engage in caregiving. Additionally, 47.14% of the participants were male, and 72.35% of the respondents were married, indicating a majority of the sample had a spouse. In terms of education, 39.14% of respondents were uneducated, 48.79% had completed elementary school, and 12.07% had received secondary or higher education, highlighting the generally low educational attainment among rural older adults in China. Lastly, 47.05% of the respondents reported drinking, while 45.03% reported smoking, with relatively balanced distributions of smoking and drinking behaviors among the participants.

**Table 2:** Characteristics of the study population (*N* = 4,366).

Variables		Frequency	Percentage (%)
Caring for Grandchildren	Provide	2,960	67.80
	Not provided	1,406	32.20
Gender	Male	2,058	47.14
	Female	2,308	52.86
Marital status	Married	3,159	72.35
	Divorced\widowed\never married	1,207	27.65
Education	Did not go to school	1,709	39.14
	Elementary school	2,130	48.79
	Middle school	435	9.96
	High school or above	92	2.11
Drinking	Yes	2,054	47.05
	No	2,312	52.95
Smoking	Yes	1,966	45.03
	No	2,400	54.97

### 4.2 Correlation and characteristics of main variables

Table 3 presents the descriptive statistics and correlations among depression, functional abilities, and caring for grandchildren. Depression is positively correlated with functional abilities (*r* = −0.237, *p* < 0.001), suggesting that better functional abilities are associated with lower levels of depression. The relationship between depression and caring for grandchildren is weak and not statistically significant (*r* = −0.057), indicating little direct association. Additionally, functional abilities and caring for grandchildren are significantly correlated (*r* = 0.117, *p* < 0.001), implying that those who care for grandchildren tend to have higher functional abilities. A positive correlation was observed between functional abilities and caregiving (*r* = 0.117, *p* < 0.001), suggesting that those with better functional abilities were more likely to take care of grandchildren. While this correlation is statistically significant, the magnitude is relatively low, and collinearity diagnostics (VIF values) were within acceptable limits, indicating that multicollinearity is unlikely to bias the regression results. Nonetheless, this relationship should be interpreted with caution, and future research could apply structural equation modeling or other approaches to better disentangle these associations. The skewness and kurtosis values for all variables are within acceptable ranges, confirming that the distribution is appropriate for further analysis.

**Table 3:** Correlation and characteristics of the main variables.

Variables	Depression	Functional abilities	Caring for grandchildren
Depression	1.000		
Functional abilities	−0.237***	1.000	
Caring for grandchildren	−0.057	0.117***	1.000
Range	0–30	0–33	0–1
Mean	9.31	28.1	0.68
SD	1.45	1.98	0.86
Skewness	1.63	−0.23	−0.61
Kurtosis	2.81	4.69	3.09

*p < 0.05, **p < 0.01, ***p < 0.001.

### 4.3 Moderating model

Table 4 presents the results of the full sample analysis on the effect of functional abilities on depression among older adults in rural China. Model 1 includes control variables and the moderating variable (caring for grandchildren), allowing us to observe the direct effect of grandchild caregiving on depression. In Model 2, functional abilities were added, and its effect on depression is highlighted. Model 3 incorporates the interaction term between functional abilities and caring for grandchildren to assess the moderating role of caring for grandchildren.

**Table 4:** Regression analysis of the moderating effect of caring for grandchildren.

Variables	Model 1		Model 2		Model 3	
	*B*	Beta	*B*	Beta	*B*	Beta
Female (ref. Male)	0.203***	0.152	0.177***	0.151	0.177***	0.151
Age	−0.007***	−0.050	−0.009***	−0.072	−0.009***	−0.072
Divorced\widowed\never married (ref. Married)	0.116***	0.077	0.118***	0.079	0.119***	0.079
Education	−0.033**	−0.036	−0.022	−0.024	−0.022	−0.024
Drinking (ref. Not Drinking)	0.008	0.006	0.006	0.005	0.004	0.003
Smoking (ref. Not Smoking)	0.032	0.023	0.028	0.020	0.028	0.020
Personal income	−0.027***	−0.100	−0.023***	−0.085	−0.023***	−0.084
Caring (ref. Not Caring)	−0.023	−0.016	−0.010	−0.007	−0.005	−0.003
Functional abilities			−0.402***	−0.219	−0.423***	−0.230
Functional abilities × Caring					−0.203***	−0.047
Constant	1.290***		2.630***		2.690***	
*R* ^2^	0.047		0.094		0.096	
Adj. *R*^2^	0.046		0.092		0.094	
△*R*-squared	–		0.046***		0.002***	
*F*	27.07***		49.89***		45.98***	

Caring means caring for grandchildren. *p < 0.05, **p < 0.01, ***p < 0.001.

The results from Model 1 indicate the following for the control variables: (1) gender—women are more likely to experience depression compared to men; (2) age—depression levels in rural older adults decrease with age; (3) marital status—divorced, widowed, or never-married individuals are more prone to depression than those who are married; (4) education—higher education levels are negatively associated with depression, suggesting that the more educated the individual, the lower their depressive symptoms; (5) smoking and drinking—neither variable had a significant impact on depressive symptoms; (6) personal income—higher income is associated with lower levels of depression.

In Model 2, the addition of functional abilities reveals a negative correlation with depression, where an increase in functional abilities corresponds to a decrease in depression levels. Finally, Model 3 examines the moderating role of caregiving for grandchildren. The interaction between functional abilities and caregiving for grandchildren (*β* = −0.203, *p* < 0.001) is significant, demonstrating that caring for grandchildren moderates the effect of functional abilities on depression. As shown in Figure 2, depression decreases as functional abilities improves, and this effect is more pronounced among those who care for grandchildren compared to those who do not. This indicates that caregiving for grandchildren can moderate the relationship between caring for grandchildren and depressive symptoms in rural older adults.

**Figure 3:**
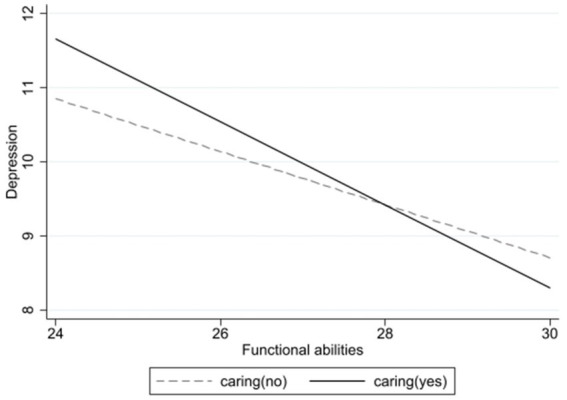
Moderating effects of caring for grandchildren on the relationship between functional ability and depression.

To further examine the moderating role of caregiving, we conducted a stratified analysis by splitting the sample into high and low functional ability groups based on the median score. Among participants with higher functional ability, caregiving for grandchildren was significantly associated with lower depression levels (*β* = −0.137, *p* < 0.01). However, among those with lower functional ability, caregiving did not have a statistically significant effect on depression. This suggests that the mental health benefits of caregiving are more evident in older adults with greater functional independence (Figure 3).

## 5 Discussion

This study examined the effect of functional abilities on depressive status in rural aged parents through regression analysis based on data from CHARLS2018 and verified the moderating effect of caring for grandchildren using hierarchical regression analysis. The results of the regression analysis of the sample indicated that the functional abilities of rural aged parents were negatively associated with depression level, H1 was supported; and the moderating effect of caring for grandchildren was also verified in the analysis of this study, H2 was supported.

In this study, higher functional abilities among rural older adults were significantly associated with lower levels of depression, consistent with previous findings (45–47). This suggests that enhanced functional capacity may serve as a protective factor against depressive symptoms in this population. The risk of functional impairment increases significantly with age, affecting abilities such as household chores, traveling, and shopping (48). functional impairment can substantially reduce an individual’s sense of self-control and self-care, posing a serious threat to independent living for older adults. Rapid functional decline may even result in feelings of hopelessness about maintaining independence (49). As older adults lose their ability to perform ADLs and IADLs, they are unable to maintain appropriate social roles, which disrupts their daily activities and fosters a psychological environment conducive to the development of depressive and anxiety symptoms (24). Impaired functional abilities also diminish opportunities for social interaction, significantly impacting their quality of life. A community-based cross-sectional study found that functional impairment was strongly associated with depressive symptoms, with older adults experiencing such impairments at a higher risk of depression (50). Similarly, a Chinese prospective cohort study revealed that physical limitations significantly increase the likelihood of depressive symptoms in aged parents (51). Ormel et al. (52) summarized the relationship between daily activities and depression as follows: first, limitations in activities of daily living and depressive symptoms form a positive feedback loop—decreased mobility leads to depression, and depression, in turn, exacerbates mobility loss. Second, depression exerts a weak but delayed negative effect on mobility. Third, the negative impact of reduced daily activities on depression is both rapid and strong.

In addition, this study analyzes the moderating role of caring for grandchildren in the impact of functional abilities on depression. The mechanism of the effect of caring for grandchildren on depression has been confirmed by many studies (53, 54), and based on these studies, this paper confirms that caring for grandchildren mitigates the effect of changes in functional abilities on the degree of depression. The findings suggest that rural older adults who provide care for grandchildren with higher functional abilities have more significant decreases in depression levels compared to those who do not provide care for their grandchildren. Based on the above findings, caring for grandchildren as an informal task can mobilize older adults in various ways, thus slowing down their depressive state. After retirement, the social roles of older adults change, the focus of life shifts from work to family, and family relationships gradually become the main source of well-being for older adults (55). In addition, the diminishing mobility that accompanies aging may cause self-doubt and a sense of uselessness in older adults, and at this time older adults desire care and encouragement from others, and active communication and emotional interaction between grandchildren and older adults can alleviate their loneliness and reduce their fear of aging, thus alleviating depression and improving their mental health.

This study also examines the moderating role of caregiving for grandchildren in the relationship between functional abilities and depression. The impact of caregiving on depression has been well-documented in several studies (53, 54), and based on these findings, this research confirms that caregiving moderates the effect of functional abilities changes on depression severity. The results indicate that rural older adults with higher functional abilities who provide care for their grandchildren experience more significant reductions in depression compared to those who do not engage in caregiving. These findings suggest that caregiving, as an informal activity, can actively engage older adults, reducing their depressive symptoms. After retirement, older adults experience shifts in social roles, with family life becoming their primary source of fulfillment (55). As mobility declines with age, feelings of self-doubt and uselessness may arise, creating a need for support and validation. In such situations, emotional interaction and communication between grandparents and grandchildren can alleviate loneliness and reduce fear of aging, ultimately improving mental health and alleviating depressive symptoms (56).

One limitation of this study is its cross-sectional design, which restricts causal inference regarding the relationship between functional abilities, caregiving, and depression. Future research should adopt longitudinal designs to explore the temporal sequence of these variables and determine whether functional decline precedes depression or vice versa. Additionally, caregiving for grandchildren was operationalized as a binary variable (yes/no), which may oversimplify the caregiving experience. Future studies should incorporate caregiving intensity, duration, frequency, and perceived burden to better capture its heterogeneous impact. Moreover, while this study controlled for several key sociodemographic variables, other influential factors such as chronic illness, social support networks, and caregiving satisfaction were not included and may have introduced residual confounding. The weak direct correlation between caregiving and depression also suggests that caregiving may not independently predict depressive symptoms but may interact with functional ability to shape mental health outcomes. Finally, this study focused exclusively on rural older adults, which may limit the generalizability of the findings to urban populations where caregiving roles and intergenerational expectations differ. Future research should conduct comparative analyses across rural and urban contexts to explore the influence of sociocultural factors on intergenerational caregiving and psychological outcomes.

## 6 Conclusion

This study demonstrates that while caregiving for grandchildren does not directly reduce depression among rural older adults, it significantly moderates the relationship between functional abilities and depressive symptoms. Older adults with higher functional capacities benefit more from caregiving roles, experiencing lower levels of depression. These findings highlight the protective effect of intergenerational engagement in the context of declining physical health. However, the cross-sectional nature of the data, the simplified measurement of caregiving, and the rural-only sample call for caution in generalization. Future research should employ longitudinal data, include detailed measures of caregiving intensity and burden, and compare rural and urban contexts to better understand how caregiving interacts with aging trajectories and mental health.

## Data Availability

Publicly available datasets were analyzed in this study. This data can be found at: https://charls.pku.edu.cn/.
